# Different Location Sampling Frequencies by Satellite Tags Yield Different Estimates of Migration Performance: Pooling Data Requires a Common Protocol

**DOI:** 10.1371/journal.pone.0049659

**Published:** 2012-11-14

**Authors:** Alessandro Tanferna, Lidia López-Jiménez, Julio Blas, Fernando Hiraldo, Fabrizio Sergio

**Affiliations:** Department of Conservation Biology, Estación Biológica de Doñana, Consejo Superior de Investigación Científica, Sevilla, Spain; Norwegian Polar Institute, Norway

## Abstract

**Background:**

Migration research is in rapid expansion and increasingly based on sophisticated satellite-tracking devices subject to constant technological refinement, but is still ripe with descriptive studies and in need of meta-analyses looking for emergent generalisations. In particular, coexistence of studies and devices with different frequency of location sampling and spatial accuracy generates doubts of data compatibility, potentially preventing meta-analyses. We used satellite-tracking data on a migratory raptor to: (1) test whether data based on different location sampling frequencies and on different position subsampling approaches are compatible, and (2) seek potential solutions that enhance compatibility and enable eventual meta-analyses.

**Methodology/Principal Findings:**

We used linear mixed models to analyse the differences in the speed and route length of the migration tracks of 36 Black kites (*Milvus migrans*) satellite-tagged with two different types of devices (Argos vs GPS tags), entailing different regimes of position sampling frequency. We show that different location sampling frequencies and data subsampling approaches generate large (up to 33%) differences in the estimates of route length and migration speed of this migratory bird.

**Conclusions/Significance:**

Our results show that the abundance of locations available for analysis affects the tortuosity and realism of the estimated migration path. To avoid flaws in future meta-analyses or unnecessary loss of data, we urge researchers to reach an agreement on a common protocol of data presentation, and to recognize that all transmitter-based studies are likely to underestimate the actual distance traveled by the marked animal. As ecological research becomes increasingly technological, new technologies should be matched with improvements in analytical capacity that guarantee data compatibility.

## Introduction

Scientific progress depends heavily on the efficacy of methodological tools and their repeatability and compatibility across studies. Migration research began with the simplest possible methodology, by observation, and has progressed over the past decades through the development of progressively more sophisticated techniques, recently culminating in the deployment of miniature satellite-transmitters to track individuals during their journey [1]–[3]. Such electronic tracking methods are rapidly diversifying into a multitude of potential models and options, such as VHF telemetry, Argos satellite telemetry, GPS tracking, and GPS/GSM devices [4]–[7]. In turn, these allow differential abundance and precision of the recorded parameters, which can often be influenced by the study design, i.e. by how the researcher plans the set-up of the remote sensor. Thus, the last few years have witnessed a proliferation of tracking studies, documenting migratory displacements of thousands of kilometres in greater detail than ever before [8], [9]. Although electronic tracking has clearly and profoundly transformed this field of science [10], such technology comes with inherent errors and biases (e.g. spatial precision afforded by different devices, or abundance of locations imposed by the researcher's set-up of the tag).

In studies of long-distance migration, these errors may have relatively limited importance for the simple visualization of the overall migration track [11], [12]. However, many behavioural studies focus on more detailed aspects of navigation, such as travelling distance, speed and direction, or smaller-scale movements [12]–[15]. Thus, while early-generation satellite transmitters stimulated enormous advancements in the study of migration, these initial tags employed relatively few locations of coarse accuracy, obtained exclusively through triangulation by Argos satellites (hereafter “Argos locations”). The fact that errors were often larger than several hundred meters (e.g. [16]), or that discontinuous reception caused several consecutive migration-days with no data (e.g. [17]) precluded the exploration of small-scale ecological questions.

More recently, the advent of tracking devices incorporating Global Positioning Systems (GPS) has further revolutionized this area of research, by providing relatively large and consistent numbers of locations (hereafter “GPS locations”) of much higher and systematic precision (spatial errors ∼15 m). Also, because GPS data are typically stored and downloaded in packages of multiple days/months of data at a time, this typically guarantees a more thorough and abundant sampling of migration behaviour. However, because GPS tags can still be placed only on relatively heavy organisms (minimum of ∼1 kg in weight), non-GPS satellite tags (hereafter “Argos tags”) continue to be widely employed and their performance (location abundance and precision) is constantly improving [18], [19].

The simultaneous use of different methods, their constant refinement, and the differential device set-up imposed by scientists lead to the coexistence of descriptive datasets incorporating differential errors and accuracies [20], [21]. This may generate impediments in using such studies in meta-analyses to uncover emergent generalities. The latter will be soon important in such a young field of research, still dominated by simple descriptions of movement performance largely describing migrations of individual species. For example, descriptive data on migration speed, length and timing are already available for dozens of raptor, seabird and Ciconiiform species, but pooling them in a single review may prove challenging. This is mainly because: (i) researchers may set-up their satellite tags in different ways, resulting in differential location sampling regimes; (ii) studies may employ different types of devices, such as Argos or GPS tags; (iii) Argos locations are further classified into various levels of estimated error (location class: “LC”, details in Methods), with different studies incorporating different thresholds of LC acceptance (Table S1); and (iv) Argos has recently implemented a new location processing algorithm (Kalman filtering) that improved the frequency and spatial error of Argos locations [16], [18]. This will further increase the across-study heterogeneity in the quality of the source data. While the issue of the actual spatial accuracy of locations has been addressed by various authors [6], [12], [22]–[24], we are not aware of broader assessments of methodological compatibility, except for the recent paper by Rowcliffe et al. [25] on non-migratory mammals. In particular, here we place emphasis on the biases in migration-performance estimation produced by the number of locations employed to delineate a migration track (hereafter “location frequency” or “location abundance”). In general, analyses based on higher location frequency could be logically expected to generate more tortuous movement-paths and thus yield larger estimates of distance travelled. This aspect has been well known since the 1980s [26]–[28] but continues to be a widely overlooked issue in movement ecology in general, as well pointed out and reviewed by Rowcliffe et al. [25]. The latter authors recently provided an in-depth demonstration of dramatic biases caused by differential radio-tracking sampling regimes in estimations of the actual distance travelled during their daily wanderings by terrestrial mammals which were year-round residents in a tropical forest. To our knowledge, analyses on the same issue have not been conducted for animals during migration.

In conclusion, a rapidly increasing number of satellite-tracking studies, the above described heterogeneity in methodologies and a growing need for meta-analyses call for an urgent assessment of the compatibility of datasets based on different location sampling regimes or different analytical methods. To provide such an evaluation, we used data from 36 Black kites *Milvus migrans* marked with Argos/GPS satellite transmitters to test the hypothesis that route length and speed of migration differ between differential regimes of location sampling frequency and across different thresholds of acceptance of Argos classes of location accuracy (i.e. across different data subsampling approaches). The final aims of the study were to: (1) test whether data based on different sampling frequencies and subsampling approaches are compatible; and (2) seek potential solutions that may enhance compatibility and enable eventual meta-analyses. Here, our objective is not to criticize previous studies, but to stimulate awareness about future biases in data comparison.

## Results

**Figure 1:**
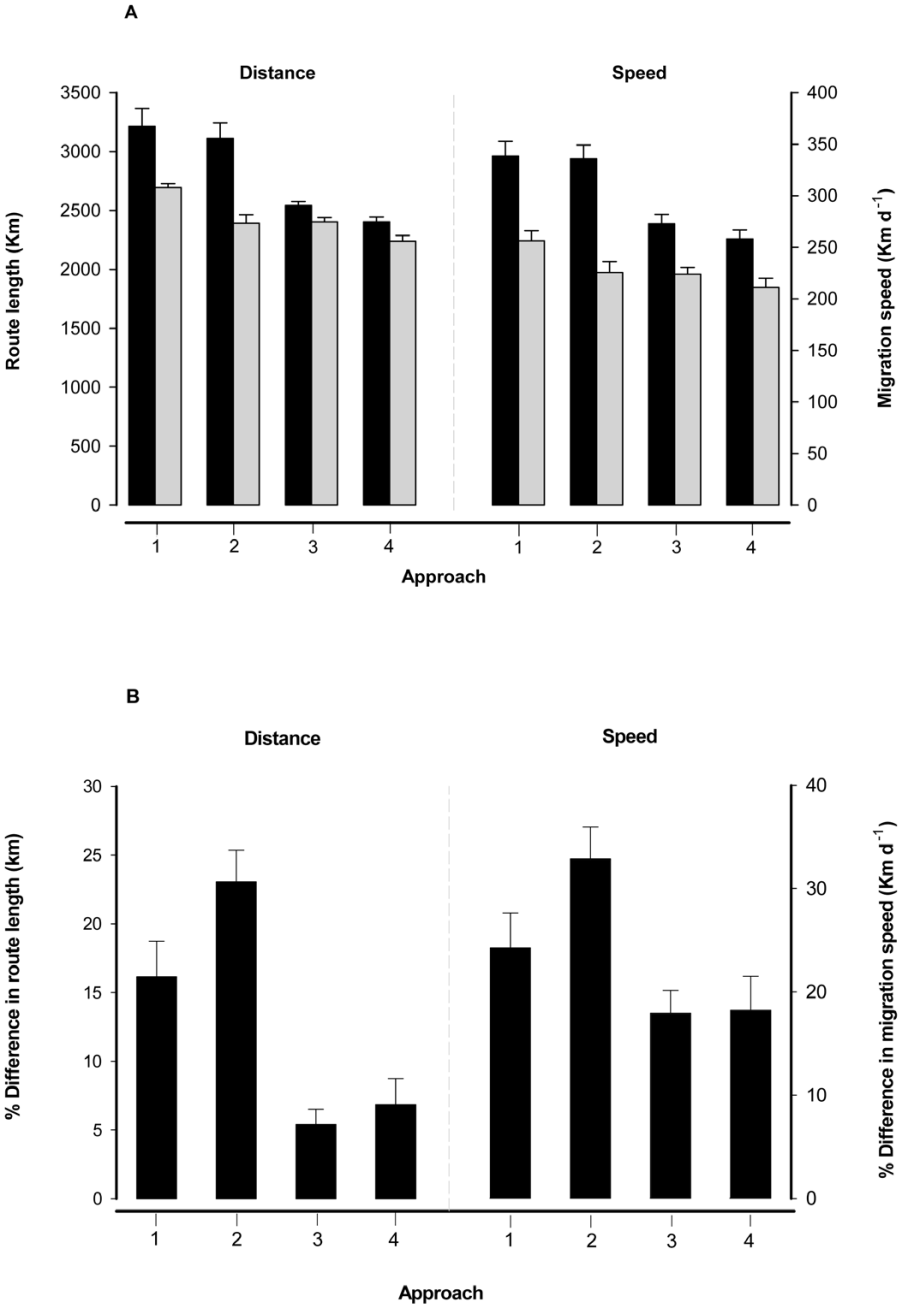
Mean migration route length and speed (A) of 36 Black Kites simultaneously marked with an Argos (grey bars) and GPS (black bars) satellite-tracking device, and the percentage difference between the two types of device (B), according to four different ways to subsample the locations for analysis. The percentage difference was calculated for each individual as “[route length (or speed) of GPS tag – route length (or speed) of Argos tag]/route length (or speed) of Argos tag. Approach 1: all available locations employed for analysis; Approach 2: all locations except nocturnal ones; Approach 3: a single diurnal location; Approach 4: a single nocturnal location (see Methods for further details). Error bars represent 1 SE.

Data were available for 3312 satellite locations, generating 72 migration routes (36 Argos and 36 GPS tracks). There were substantial differences in route length and speed of migration between GPS and Argos tags for all the four examined approaches (Table 1). In all cases, the route lengths and speeds calculated through GPS tags (i.e. through a regime of higher location sampling frequency) were significantly larger than those based on Argos tags. Depending on the data subsampling approach considered, kite migration routes were 5 to 23% longer and their speed 18 to 33% faster when assessed by GPS than by Argos locations (Fig. 1A).

**Table 1:** Paired t-tests comparing the route length and migration speed of 36 Black Kites equipped with Argos vs GPS satellite-tracking devices for each of four data subsampling approaches (details in methods).

Variable and Approach	Mean ± SE (n, min, max^a^)		t	P
	Argos	GPS		
**Route length (km):**				
(Approach 1) All locations	2695±33.6 (21.2,9,37)	3214±151. (70.7,52,129)	3.763	0.001
(Approach 2) All locations except nocturnal ones	2394±70.6 (17.2,8,31)	3112±132.2 (51.7,37,89)	6.931	<0.0001
(Approach 3) One location per day	2405±34.8 (4.1,2,6)	2542±33.9 (10.0,6,17)	3.704	0.001
(Approach 4) One location per night	2240±48.4 (3.7,2,6)	2404±39.6 (9.1,5,16)	2.589	0.014
**Migration speed (Km d^−1^):**				
(Approach 1) All locations	256±9.7 (21.2,9,37)	338±14.4 (70.7,52,129)	5.542	<0.0001
(Approach 2) All locations except nocturnal ones	226±10.6 (17.2,8,31)	336±13.5 (51.7,37,89)	8.682	<0.0001
(Approach 3) One location per day	224±6.7 (4.1,2,6)	273±8.9 (10.0,6,17)	7.021	<0.0001
(Approach 4) One location per night	211±8.8 (3.7,2,6)	258±8.6 (9.1,5,16)	4.671	<0.0001

a n = mean number of locations/tag on which the estimate is based; min = minimum number of locations/tag on which the estimate is based; max = maximum number of locations/tag on which the estimate is based.

Type of location (Argos vs GPS), type of subsampling approach (Approach 1–4, see Methods for details) and their interaction entered the most competitive LMM, both when using route length or migration speed as dependent variables (Table 2). These models confirmed that the GPS route length and speed were larger than the Argos ones (Table 3): on average, there was a difference of 518.7±93.5 km in route length (*t* = 5.0, df = 245, *P* = 0.00001) and of 82.1±10.6 Km d^−1^ in speed (*t* = 7.3, df = 245, *P* = 0.00001) between Argos and GPS tags. However, the magnitude of such differences also depended on the approach used to subsample the data (Table 3 and Fig. 1B), being greatest for Approach 2 and minimum for Approaches 3 and 4 (Fig. 1B).

**Table 2:** Linear mixed models (LMMs) of route length (a) and migration speed (b) by satellite-tracked Black Kites on type of tagging device (“Device Type”: Argos vs GPS) and data subsampling approach (“Approach”: 1 to 4, see Methods).

Variables	Model	df	AIC	Likelihood ratio	*P*
**(a) Response variable: Route length (km)**					
Device Type^a^+Approach^b^+Device Type^a^ * Approach^b^	1	10	−789.6		
Device Type^a^+Approach^b^	2	7	−768.8	26.8	<0.0001
Device Type^a^	3	4	−672.9	101.8	<0.0001
Approach^b^	4	6	−704.4	35.5	<0.0001
Intercept-only model	5	3	−628.8	81.7	<0.0001
**(b) Response variable: Migration speed (km d^−1^)**					
Device Type^a^+Approach^b^+Device Type^a^ * Approach^b^	1	10	−628.6		
Device Type^a^+Approach^b^	2	7	−614.5	20.2	0.0002
Device Type^a^	3	4	−546.9	73.6	<0.0001
Approach^b^	4	6	−467.7	75.1	<0.0001
Intercept-only model	5	3	−430.3	43.4	<0.0001

a Dychotomic variable: Argos tag vs GPS tag.

b Categorical variable: 1 = all locations employed for analysis (Approach 1); 2 = all locations except nocturnal ones (Approach 2); 3 = a single diurnal location employed for analysis (Approach 3); 4 = a single nocturnal location (Approach 4). See Methods for further details.

**Table 3:** Parameter estimates and details of the most competitive models of Table 2, depicting the relationship between the route length (1) or migration speed (2) of 36 satellite-tagged Black Kites and Device Type (Argos vs GPS) and data filtering Approach (Approach 1–4).

Most competitive model	Parameter estimate ± SE	t-value	*P*
**(1) Response variable: route length (km)**			
Device Type	518.7±93.5	5.0	<0.0001
Approach 2	−300.9±93.5	−4.5	<0.0001
Approach 3	−289.8±93.5	−3.9	0.0001
Approach 4	−454.9±93.5	−6.5	<0.0001
Device Type *Approach 2	198.8±132.2	2.6	0.0112
Device Type *Approach 3	−381.4±132.2	−2.2	0.0307
Device Type * Approach 4	−354.2±132.2	−1.7	0.0883
Intercept	2694.9±132.2	302.9	<0.0001
**(2) Response variable: migration speed (km d^−1^)**			
Device Type	82.1±10.6	7.3	<0.0001
Approach2	−30.9±10.6	−3.8	0.0002
Approach3	−32.4±10.6	−3.4	0.0009
Approach4	−45.2±10.6	−5.4	<0.0001
Device Type * Approach 2	28.2±15.0	2.6	0.0108
Device Type * Approach 3	−33.1±15.0	−1.5	0.1321
Device Type * Approach 4	−35.1±15.0	−1.2	0.2502
Intercept	256.4±15.0	142.5	<0.0001

## Discussion

**Figure 2:**
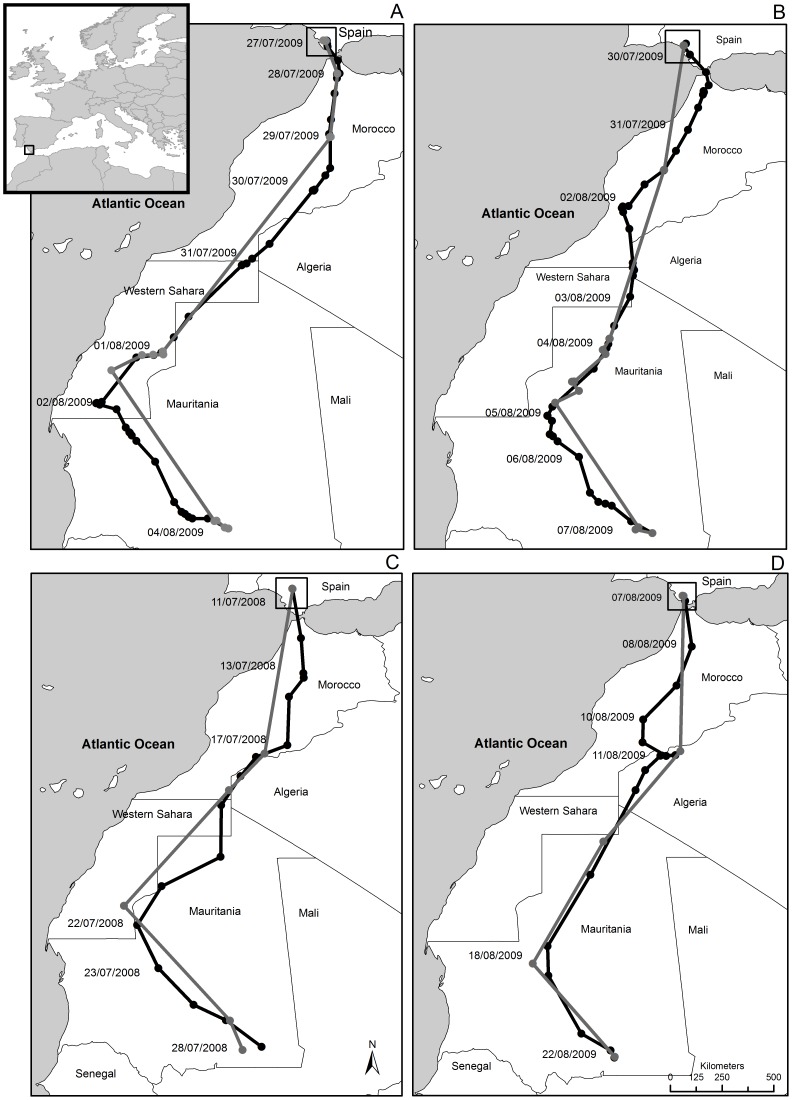
Example of migration paths of four different individual Black Kites as assessed for each one by a GPS tag (black line) and an Argos tag (grey line employing Argos Location Classes 0–3). In panels A and B, locations were subsampled using Approach 1 (all locations used for analysis), while in panels C and D, locations were subsampled using Approach 3 (one location per day), Kites were tagged in Doñana National Park (south-western Spain): its location in Europe is portrayed in the inset. The grey, Argos routes are systematically based on fewer locations and thus composed of fewer straighter segments, resulting in shorter, less tortuous routes and lower migration speeds.

Our results show that descriptors of migration routes and performance vary with location sampling frequency and with the type of analytical approach used to subsample the data. Furthermore, the variation caused by the sampling artifact can be dramatic. In our case, usage of a lower sampling regime (compatible with configurations or irregular data reception of previous studies, especially of first-generation tags) generated a 5–33% difference in migration estimates (Table 1). Interestingly, while most previous studies have focused heavily on the spatial accuracy and reliability of Argos-locations [12], [22], [23], [29], [30], our results are essentially generated by differences in the abundance of locations employed to estimate the migration descriptors (see below). This could be essentially considered as a bias caused by the “experimental design” of the study (set-up of location frequency requested by the researcher or allowed by the device). In our study, given the planned set-up of the devices, GPS tags systematically afforded a greater number of locations, which led to more tortuous and curvilinear, longer routes than the Argos ones, characterized by fewer, straighter segments (see examples in Fig. 2). In turn, longer routes traveled in the same time-span generated faster migration speeds. In extreme situations, Argos locations were so infrequent that they generated “virtual” routes, such as sea-crossings outside the Strait of Gibraltar, known to be false paths when compared to GPS data (Fig. 2B). Similar tracks, with artificial straight lines connecting locations distanced several days apart, have been previously reported by other authors (e.g. [17], [31]).

For the same reason, subsampling approaches that yielded larger numbers of locations for analysis generated longer routes and faster speeds (e.g. Approaches 1 and 2 in Fig. 1). This implies that subsampling approaches based on a single location per day or night will render migration descriptors more comparable across studies or devices that employ different sampling regimes (see Approach 3 and 4 in Fig. 1B), provided that all datasets are subsampled in the same way (i.e. reduced to one location per day). This is because, in our case, such an approach helped to equalize more the number of locations obtained with the two types of tags, although Argos tracks were of course still based on fewer days with valid locations than GPS migration tracks (e.g. Fig. 2, panels C and D). On the contrary, approaches that maximize the number of locations available for analysis will incorporate higher inherent relative errors, but will yield larger absolute estimates of migration routes and speed, which are closer to the real ones (see Approach 1 and 2 in Fig. 1B). All the above implies that researchers will be confronted with a trade-off between: (1) data subsampling approaches that yield descriptors that are surely inaccurate but as close as possible to the real estimates; or (2) descriptors that are more inaccurate in absolute terms but more comparable across studies and devices. The best approach will depend on the final aim of a potential study or meta-analysis. For example, an author interested in the simple, crude delineation of a migration route may employ whatever method that maximizes the number of locations available, while an author testing the effect of body size on migration speed across species may be more interested in data comparability across devices (i.e. Approach 3 and 4) rather than estimation of the absolute route length of each species. On the contrary, a meta-analysis focusing on the energetics of migration may necessitate estimates of route length and speed as close as possible to reality (i.e. Approach 1 and 2).

Overall, we stress that any factor causing variation in location frequency may undermine the comparability of quantitative migration descriptors. Such problem will apply to all scales of comparison. For example, long-term marking projects which employed progressively more refined tags yielding progressively higher location frequency will suffer of within-study heterogeneity [32], while meta-analyses of the migration literature will unavoidably mix early and last generation tags that typically allowed different regimes of location frequency. Such overall problem is likely to persist for decades to come. For example, GPS tags that allow locations every few minutes are becoming available in the market in ever lighter weight, which will soon generate analyses based on hundreds-fold differences in location abundance compared to current tags. On the other hand, in 2011 CLS-Argos implemented a new algorithm (Kalman filtering) that substantially improved the frequency and accuracy of Argos locations [18], [19]. As a result, the literature will increasingly incorporate even more heterogeneous estimates based on Argos traditional locations, Argos Kalman-filtered locations, and GPS tags with profoundly different set-up configurations, further exacerbating the compatibility issue.

The compatibility concern we express here has been long recognized [21] and is complemented by similar views expressed by Rowcliffe et al. [25] in a detailed study on the estimation of the distance travelled by non-migratory mammals. These authors showed similarly dramatic biases caused by different regimes of sampling frequency. They further showed that measurement of the real path travelled by a target animal would require a probably unrealistic sampling regime of one tracking-location every few seconds. This implies that all researchers should be aware that they are always underestimating the real distance travelled. Unfortunately, despite the potential magnitude of the bias involved, the problem of location abundance seems to have been largely overlooked in migration studies as well as in general movement ecology [25].

The above concerns call for an urgent need of agreement over a protocol of analysis and presentation of descriptive satellite-tracking data. A rapid look at the literature makes it clear that such agreement is far from present, with a bewildering array of approaches simultaneously undertaken (Table S1). Ideally, the solution would be availability of original datasets through electronic storage initiatives, such as Movebank (http://www.movebank.org/). However, it seems unlikely that all authors will routinely offer their raw data to the scientific community, for example because they will want to publish them before making them available to others. In such cases, we suggest two procedures that could maximize the chance that future authors will be able to access previously published estimates in a meaningful way. (1) Firstly, researchers should provide descriptive estimates based on all the four filtering approaches described here (e.g. see an example in Table 1). Given that most satellite-tracking studies are still descriptive in nature, we believe that adding some further descriptive statistics would not represent an excessive burden for the authors. (2) Secondly, each migration estimate should be accompanied by an estimate of the number of locations used to calculate it (e.g. see an example Table 1). Inclusion of such information would allow authors of meta-analyses to control statistically for among-study differences in location-frequency (e.g. by fitting location-frequency as a random factor in mixed effects models) or to discard incompatible estimates that would add excessive statistical noise to the analysis. Ideally, we incite authors of previously published satellite-tagging studies to make their descriptive estimates available again in a more accessible format, for example as short data-papers. As in many cases satellite-tracking is accomplished by specialized research teams that sequentially mark various species, they could provide in a more useful format all the already published descriptive estimates for these species in a single data paper.

Finally, our analysis focused on animals that migrated through a relatively direct, straight route from a starting point A to an endpoint B. However, several studies have reported more complex and convoluted migrations where a starting and endpoint are difficult to identify, or with so many interruptions and stopovers that calculating an overall route length and speed is difficult or impossible [33]–[37]. However, even in these cases, authors often report descriptive estimates, such as speed, for portions of the journey [17], [36], [38], [39]. The biases that we outlined for more traditional migrations would equally apply to such estimates (see also [25]). In conclusion, as ecological research grows increasingly technological [40], [41], it will be fundamental that technological refinements are closely tracked by improvements in analytical capacity that ensure data compatibility.

## Methods

### Study area, model species and transmitter data

In 2008 and 2009, we equipped 36 Black kites with satellite transmitters in Doñana National Park (south-western Spain) [42]. The Black kite is a medium-sized migratory raptor which breeds in Europe from March to August [43] and spends the rest of the year in its wintering grounds of the Sahel region of Africa. We used satellite tags “solar/Argos GPS PTT 100” (22 g) manufactured by Microwave Inc. (http://www.microwavetelemetry.com). Each tag incorporated a GPS receiver and was fitted to the birds using a Teflon harness [44]. Thus, the tags simultaneously yielded both Argos and GPS locations for each individual. We programmed the platforms to transmit every three days and to obtain 8 GPS fixes every day between 06:00 and 24:00 hours (the maximum number suggested by the manufacturer). This implies that GPS locations were available for every day of the migration, while Argos locations were available for a full day every three days, resulting in a lower sampling frequency. Such level of location abundance is common in the literature, where holes in the data series of up to six consecutive days have been reported (e.g. [17], [45], [46]). Irregular sampling can be caused by funding limitations (higher frequency implies higher Argos-costs), failure of satellite coverage, or weaker satellite coverage of certain areas of the earth (e.g. portions of the Mediterrenean [16]). Therefore, we considered the two types of locations (Argos and GPS) as treatments based on differential regimes of sampling frequency. Argos locations, calculated on the basis of the Doppler Effect, were downloaded from the Argos web site by means of the PRV command. GPS positions were acquired through the PRV/A-DS command, following the methodology indicated by Soutullo et al. [24].

Argos locations are provided by Argos to users with an associated estimate of accuracy (location class: “LC”), based on the quality of signal detection by the Argos satellites. Thus, LCs 3,2,1,0 respectively correspond to spatial errors of <250, 250–500, 500–1500 and >1500 m [16], while LC A and B correspond to locations of unknown errors. Most previous studies discard LCs A and B and employ a variety of approaches to subsample the Argos locations used for statistical analysis (Table S1). Four main approaches are reported in the literature: (Approach 1) inclusion of all the available locations; (Approach 2) exclusion of all nocturnal locations; (Approach 3) restriction to a single diurnal location per 24 hours; and (Approach 4) employment of a single nocturnal location per 24 hours.

To compare Argos locations with GPS ones in our dataset, we: (1) subsampled Argos locations according to the four approaches explained above; (2) subsampled GPS locations in the same manner in order to make them comparable to the Argos dataset; (3) on the basis of each of the four approaches, we estimated the outward, autumn migration route, length and speed of each kite individual using either Argos or GPS locations; and (4) compared the migration estimates between Argos and GPS locations for each of the four subsampling approaches. Thus, for example, the migration speed obtained for a kite through Approach 3 from Argos locations was compared with the migration speed obtained for the same individual through Approach 3 for GPS locations.

Further, more detailed subsampling adjustments of the datasets were implemented to conform to standard methodologies employed in previous studies. Thus, (A) to calculate the distance and speed using Approaches 3 and 4 based on a single location, we selected only the Argos location of highest accuracy; (B) for Approach 3 based on a single diurnal point, when several locations of equal accuracy were available during daylight hours, we used the one closest to 12:00 hours, as usually done in other studies under the assumption that birds would be more likely to perform active flying migration in that portion of the day; (C) for Approach 4 based on a single nocturnal point, the location closest to midnight was selected to conform with previous studies; and (D) all locations and especially those of lower quality (LC 0) were used only after checking their reliability via aberrant data filtering [12], [30].

The starting point and date of each migratory journey was chosen on the basis of an abrupt change in the pattern of movement by the focal individual. Similarly, an individual was considered to have ended its migration when it stopped a clearly directional, continuous and southward movement to settle in a circumscribed area (in the extreme South of Mauritania for all individuals, Fig. 2).

### Data analyses

“Route length” and “distance travelled” were equally defined as the distance covered during the migration journey, based on all available locations (i.e. including small-scale excursions for feeding, roosting etc). They were calculated on the basis of the loxodrome (rhumbline) distances covered during migration [47] using R 2.10.1 [48]. Such distances were then used to calculate migration speeds [17].

In a preliminary analysis, we used paired t-tests to compare the migration estimates within the same individual between Argos and GPS devices for the four approaches. We then refined such analyses through generalised linear mixed models (LMM; [49]). These were built by fitting distance or speed as the response variable and the following explanatory variables as fixed effects: (1) the type of tag model (Argos vs GPS tag); (2) the type of approach in subsampling the data (Approach 1, 2, 3 or 4, see above); and (3) their interaction. The identity of the 36 individuals (each one pseudo-replicated eight times in the LMMs) was included as a random factor [50]. The best model was selected among potential candidate models (based on all the combinations of explanatory variables) using Akaike's Information Criterion (AICc, [51]). Models within 2 AIC_c_ units of the best model were considered as competitive models. All LMMs were built through the *lme* function in R 2.10.1 and their assumptions were assessed graphically through diagnostic plots [49]. All tests were two-tailed, statistical significance was set at α<0.05 and all means are given ±1 standard error. When necessary, distance and speed were log-transformed to meet the assumptions of normality. We used ArcGis 9.2 [52] to map the migration routes based on Argos and GPS locations.

This study was conducted in strict accordance with the national and European legislation on animal bio-ethics. The protocol was approved by the bio-ethics evaluation CEBA-EBD_11-25 operated by the Commité Ético de Bienestar Animal (CEBA).

## Supporting Information

Table S1 Selected examples of methods used to estimate distance and speed in avian migration studies. “GPS” are tags that incorporate a GPS; “Argos” are tags that do not incorporate a GPS. LC = Argos Location Classes (which range from 0 to 3; Argos 2011).
(DOC)
