# Supplementary material for: Different Location Sampling Frequencies by Satellite Tags Yield Different Estimates of Migration Performance: Pooling Data Requires a Common Protocol
Source: PLoS One. 2012 Nov 14;7(11):e49659. doi: 10.1371/journal.pone.0049659 (PMC3498226; doi:10.1371/journal.pone.0049659)
Supplement: Table S1 — Selected examples of methods used to estimate distance and speed in avian migration studies. “GPS” are tags that incorporate a GPS; “Argos” are tags that do not incorporate a GPS. LC = Argos Location Classes (which range from 0 to 3; Argos 2011). (DOC) [file pone.0049659.s001.doc]

Table S1. Selected examples of methods used to estimate distance and speed in avian migration studies. “GPS” are tags that incorporate a GPS; “Argos” are tags that do not incorporate a GPS. LC = Argos Location Classes (which range from 0 to 3; [1]).

| **Device** | **Number of individuals tagged** | **Number of locations used for the analysis** | **Descriptors of migration employed and Notes** | **References** |
| --- | --- | --- | --- | --- |
| Argos | 18 | All validated LCs 0, 1, 2, 3 | Distance (km); speed (km d^-1^ and km h^-1^). Compare the speed estimates with data from other studies | [2] |
| Argos | 22 | One location per day | Speed (Km d^-1^) | [3] |
| Argos | 28 | Unspecified | Distance (km) and speed (Km d^-1^) | [4] |
| Argos | 2 | Only high-quality locations with a presumed error between 150 m and several kilometres | Speed (Km d^-1^), Compare the speed data with other specie | [5] |
| Argos | 11 | All validated LCs 0, 1, 2, 3 | Speed (Km d^-1^) | [6] |
| Argos | 17 | 0-15 positions during one 10 h period | Migration day, distance (Km), speed (Km d^-1^), Compare the speed data with other specie | [7] |
| Argos | 10 | All validated LCs 0, 1, 2, 3 and locations obtained < 1 h after the previous one were excluded from the analyses | Migration day, distance (Km), speed (Km d^-1^) | [8] |
| Argos | 27 | One location per night, selecting the one with highest accuracy | Speed (Km d^-1^) | [9] |
| Argos | 9 | One location per day, selecting the one with highest accuracy (lower classes used if they were the only available) | Migration day, distance (Km), speed (Km d^-1^) | [10] |
| Argos | 56 | All validated LCs 0, 1, 2, 3 | Migration day, distance (Km), speed (Km d^-1^) | [11] |
| Argos | 74 | One location per day, selecting the one with highest accuracy | Migration day, distance (Km), speed (Km d^-1^) | [12] |
| Argos | 21 | Unspecified | Speed (Km d^-1^), departure times. | [13] |
| Argos | 21 | All validated locations for LC 0 to 3 and LC A and B when location 0-3 were not available | Migration day, speed (Km d^-1^) | [14] |
| Argos | 4 | Best successive best daily location for LC 0-3 and A and successive high accuracy fixes for time periods of between 0.5 and 2 h | Daily and hourly rates of travel (Km d^-1^and Km h^-1^) | [15] |
| Argos + GPS | 16 (13 Argos + 3 GPS) | All validated locations for LC 0 to 3 for Argos device and all daily GPS location (from 05.00 to 21.00 h) | Daily distance (Km d^-1^) and speed (Km h^-1^) | [16] |
| GPS | 7 | One location per 24-h period, separated by at least 100 km from the preceding location | Migration day, speed (Km d^-1^) | [17] |
| GPS | 2 | Unspecified | Migration day, distance (Km) | [18] |
| GPS | 1 | All GPS locations once an hour from 06:00 to 22:00 every day | Speed (Km d^-1^) | [19] |

**References**

1. Argos (2011) User’s manual.: CLS/Service Argos. Toulouse, France.

2. Hake M, Kjelle N, Alerstam T (2001) Satellite tracking of Swedish Ospreys *Pandion haliaetus* : autumn migration routes and orientation. J Avian Biol 1: 47–56.

3. Thorup K, Alerstam T, Hake M (2003) Bird orientation: compensation for wind drift in migrating raptors is age dependent. Proc R Soc Lond B Biol Sci (Suppl.): 8–11.

4. Mcgrady MJ, Ueta M, Potapov ER, Utekhina I, Masterov V, et al. (2003) Movements by juvenile and immature Steller’s Sea Eagles *Haliaeetus pelagicus* tracked by satellite. Ibis 145: 318–328.

5. Trierweiler C, Koks BJ, Drent RH, Exo KM, Komdeur J, et al. (2007) Satellite tracking of two Montagu’s Harriers (*Circus pygargus*): dual pathways during autumn migration. J Ornithol 148: 513–516.

6. López-López P, Limiñana R, Mellone U, Urios V (2010) From the Mediterranean Sea to Madagascar: Are there ecological barriers for the long-distance migrant Eleonora’s falcon? Landsc Ecol 25: 803–813.

7. Strandberg R, Klaassen RHG, Hake M, Olofsson P, Thorup K, et al. (2008) Complex timing of Marsh Harrier *Circus aeruginosus* migration due to pre-and post-migratory movements. Ardea 96: 159–171.

8. Limiñana R, Soutullo A, Urios V (2007) Autumn migration of Montagu’s harriers *Circus pygargus* tracked by satellite telemetry. J Ornithol 148: 517–523.

9. Klaassen RHG, Hake M, Strandberg R, Alerstam T (2011) Geographical and temporal flexibility in the response to crosswinds by migrating raptors. Proc R Soc Lond B Biol Sci 278: 1339–1346.

10. Terraube J, Mougeot F, Cornulier T, Verma A, Gavrilov A, et al. (2011) Broad wintering range and intercontinental migratory divide within a core population of the near-threatened pallid harrier. Divers Distrib 18: 401–409.

11. Burnham KK, Newton I (2011) Seasonal movements of Gyrfalcons *Falco rusticolus* include extensive periods at sea. Ibis 153: 468–484.

12. Martell MS, Henny CJ, Nye PE, Solensky MJ (2001) Fall migration routes, timing, and wintering sites of North American Ospreys as determined by satellite telemetry. Condor 103: 715–724.

13. Fox AD, Glahder CM, Walsh AJ (2003) Spring migration routes and timing of Greenland white-fronted geese results from satellite telemetry. Oikos 103: 415–425.

14. Shamoun-Baranes J, Baharad A, Alpert P, Berthold P, Yom-Tov Y, et al. (2003) The effect of wind, season and latitude on the migration speed of white storks *Ciconia ciconia*, along the eastern migration route. J Avian Biol 34: 97–104.

15. Sittler B, Aebischer A, Gilg O (2010) Post-breeding migration of four Long-tailed Skuas (*Stercorarius longicaudus*) from North and East Greenland to West Africa. J Ornithol 152: 375–381.

16. Chevallier D, Handrich Y, Georges JY, Baillon F, Brossault P, et al. (2010) Influence of weather conditions on the flight of migrating black storks. Proc R Soc Lond B Biol Sci 277: 2755–2764.

17. Alerstam T, Hake M, Kjellen N (2006) Temporal and spatial patterns of repeated migratory journeys by ospreys. Anim Behav 71: 555–566.

18. Garcia-Ripolles C, Lopez-Lopez P, Urios V (2010) First description of migration and wintering of adult Egyptian Vultures *Neophron percnopterus* tracked by GPS satellite telemetry. Bird Study 57: 261–265.

19. Urios V, López-López P, Limiñana R, Godino A (2010) Ranging behaviour of a juvenile Bearded Vulture (*Gypaetus barbatus meridionalis*) in South Africa revealed by GPS satellite telemetry. Ornis Fennica 87: 114–118.
